# Applications of genome-scale metabolic models to investigate microbial metabolic adaptations in response to genetic or environmental perturbations

**DOI:** 10.1093/bib/bbad439

**Published:** 2023-12-02

**Authors:** Elena Lucy Carter, Chrystala Constantinidou, Mohammad Tauqeer Alam

**Affiliations:** Warwick Medical School, University of Warwick, Coventry, CV4 7HL, UK; Warwick Medical School, University of Warwick; United Arab Emirates University, Al Ain, UAE

**Keywords:** genome-scale metabolic models, metabolic adaptation, environmental variation, ‘-omics’ data, tools, simulation, emerging human pathogens, host switching

## Abstract

Environmental perturbations are encountered by microorganisms regularly and will require metabolic adaptations to ensure an organism can survive in the newly presenting conditions. In order to study the mechanisms of metabolic adaptation in such conditions, various experimental and computational approaches have been used. Genome-scale metabolic models (GEMs) are one of the most powerful approaches to study metabolism, providing a platform to study the systems level adaptations of an organism to different environments which could otherwise be infeasible experimentally. In this review, we are describing the application of GEMs in understanding how microbes reprogram their metabolic system as a result of environmental variation. In particular, we provide the details of metabolic model reconstruction approaches, various algorithms and tools for model simulation, consequences of genetic perturbations, integration of ‘-omics’ datasets for creating context-specific models and their application in studying metabolic adaptation due to the change in environmental conditions.

## INTRODUCTION

Since the first genome-scale metabolic reconstruction in 1999 for *Haemophilus influenzae* RD [1], such models have become a major approach to studying the metabolism of an organism at the systems level. The metabolic models of thousands of bacteria, and hundreds of archaea and eukaryotes have been reconstructed [2]. Genome-scale metabolic models (GEMs) computationally describe the entire set of metabolic reactions existing within an organism [1, 3, 4] and are built by integrating genome annotation and experimental data. Genome annotation quality is therefore paramount in metabolic reconstructions.

The applications for metabolic reconstructions of microbial species are wide-reaching within biology, including but not limited to the design of metabolic engineering approaches [5–7], the modeling of microbial communities [3, 8–11], identification of potential drug targets through *in silico* gene knockout experiments [12–14], studying metabolic adaptations contributing to virulence and pathogenesis [15, 16] and exploring how microorganisms reprogram their metabolism in response to environmental variation [17–19].

During their lifestyle as reproducing cells, dormant cells, commensals or pathogens, microbes encounter multiple different environmental perturbations, both externally and within a host niche, to which microorganisms must adapt accordingly. Such perturbations are often difficult to study in a laboratory, both technically and financially.

The ever-increasing availability of ‘-omics’ data for microbial species has allowed the integration of such data with GEMs to create context-specific metabolic reconstructions [20–22], detailing the metabolic adaptations occurring in an organism between two separate conditions. Analysis of such context-specific models can generate predictions of how microbes adapt their metabolism in response to environmental perturbations including nutrient limitation, pH, temperature, osmotic stress and oxidative stress. Such predictions can identify genes or pathways of interest to study further *in vitro* with the aim of identifying just how microbes alter the way they metabolize and respire in order to best survive, colonize a host, induce virulence factors, evade the host’s immune system or produce products of biotechnological interest.

### Genome sequencing and annotation

Since the late 2000s, high-throughput sequencing technologies have become more frequently applied in the biology field and have been continually developing. Since first-generation Sanger DNA sequencing technologies [23–25], next-generation sequencing (NGS) platforms have subsequently been developed with reduced sequencing costs and improved speed and quality. These platforms commonly work on the principles of creating template short DNA fragments (~600bp) followed by clonal amplification before sequencing and assembly or alignment to reference genomes [26], such as Illumina technologies [27]. The genome of most of the model organisms was sequenced using NGS technologies. Third-generation sequencing, a further development in sequencing technologies, includes the incorporation of fluorescently-labelled nucleotides before real-time sequencing of single molecules [26], such as PacBio sequencing [28]. The major challenge, however, in the NGS field is bioinformatics data analysis, for instance genome assembly and annotation. Whole genome annotation is typically a combination of homology-based methods, where the annotation is assigned to new DNA sequences based on similarity with known genes, followed by gene prediction methods, which are based on intrinsic DNA sequence signatures [29]. For prokaryotic species, annotation tools include EasyGene [30], GeneMark [31], the NCBI Prokaryotic Genome Annotation Pipeline (PGAP) [32], DFAST [33], MicrobeAnnotator [34], Prokka [35] and SNAP [36].

DFAST, MicrobeAnnotator and Prokka all utilize homology-based annotation pipelines whereby reference databases or genomes are used to identify sequence similarity facilitating the prediction of protein coding genes [33–35]. In contrast, EasyGene, GeneMark and SNAP employ intrinsic, or *ab initio*, gene prediction methods [30, 31, 36]. PGAP is unique in that it employs both homology-based and intrinsic gene prediction methods via the gene finding program GeneMarkS [32, 37]. All of the tools described above utilize a hidden Markov model as part of the annotation pipeline, with the exception of PGAP which instead employs a hidden semi-Markov model [30–36]. The outputs provided by these tools also differ, for example Prokka produces files which must be viewed in a genome browser for interpretation [35], while MicrobeAnnotator can provide graphical outputs [34]. While all these tools can be utilized for the annotation of prokaryotic genomes, GeneMark also provides species-specific software versions for even more precise and accurate annotation [31].

Due to the revolution in the sequencing field, an enormous number of species have been sequenced and annotated [38, 39]. High-quality genome sequencing and annotations are a vital starting point for the reconstruction of metabolic networks at a systems level [40], which typically starts by generating a draft automated reconstruction which is further manually curated using knowledge from several biochemical databases and the literature to fill any gaps and address the presence of dead-end metabolites.

### Reconstruction of genome-scale metabolic models

With whole genome sequencing techniques becoming more accessible, the number of sequenced genomes has been growing exponentially since the first bacterial genomes were sequenced in 1995 [41–43]. As a result, various automated model reconstruction tools have emerged. These draft reconstructions are built using a genome annotation, and therefore these drafts are only as good as the quality of the annotation.

**Figure 1:**
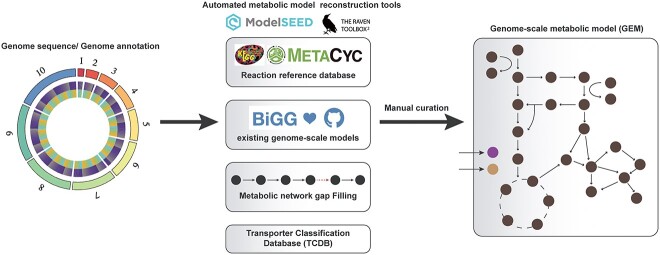
Schematic of fully functional genome-scale metabolic model reconstruction. Reconstruction of genome-scale metabolic models (GEMs) begins with the generation of an automated draft reconstruction from an inputted genome annotation. There are several tools available for the reconstruction of these draft networks. Draft reconstructions require manual curation involving gap filling and the correction of dead-end metabolites by adding in reactions detailed in biochemical reference databases, including BiGG [55], KEGG [56] and the Transporter Classification Database [57].

There are several tools available for the creation of genome-scale metabolic reconstructions. The most commonly employed of these tools are the Model SEED [44], the RAVEN toolbox [45], *merlin* [46], CarveMe [47] and Pathway Tools [48], though others do exist (Table 1). The major advantage of these new tools is that they produce simulation-ready GEMs, and some of them are also capable of implementing different analysis techniques and approaches (Table 1). The key differences among these tools include the type of input genome sequence, different reference reaction databases and various gap filling approaches. Annotated genome sequences are the required input for the RAVEN Toolbox, CarveMe and Pathway Tools, whereas Model SEED and *merlin* have inbuilt genome annotation tools (Table 1). All of these tools use a different combination of existing biochemical reference databases such as KEGG [56], MetaCyc [58], existing GEMs for other species in the BiGG database [55] and the Transporter Classification Database (TCDB) [57] (Table 1 and Figure 1). Gap filling is the most important feature for producing simulation-ready models, and each tool utilizes different approaches for this process. The Model SEED [44] and Pathway Tools [48] perform complete gap filling iteratively whereas the RAVEN Toolbox [45] identifies candidate reactions and allows the user to properly assign gene-protein-reaction (GPR) associations before filling these gaps. The CarveMe [47] tool is based on top-down model reconstruction for specific organisms, where additional media constituents are added and gap filling is performed accordingly. The *merlin* [46] tool on the other hand does not support the automated filling of gaps though it does identify these gaps in the network.

**Table 1:** Properties of automated metabolic reconstruction tools

Tool	Input	Reference databases	User platform	Network visualization	Gap filling capability	Analysis capability	Reference
Model SEED, Pathway Tools, RAVEN Toolbox	Unannotated or annotated sequence (RAST)	Model SEED, MetaCyc, KEGG	Web, GUI, MATLAB	✓	✓	✓	[44, 45, 48]
CarveMe	Unannotated sequences	BiGG	Command-line	×	✓	✓	[47]
AuReMe, FAME, GEMSiRV	Protein-coding sequences, other GEMs, KEGG Organism ID	MetaCyc, BiGG, KEGG, Model SEED	GUI, Web	✓	×	✓	[49–51]
*merlin*	Unannotated or annotated sequence	KEGG, TCDB	GUI	✓	×	×	[46]
CoReCo	Protein-coding sequences	KEGG	MATLAB	×	✓	×	[52]
AutoKEGGRec, MetaDraft	KEGG Organism ID, other GEMs	KEGG, BiGG	MATLAB, GUI	×	×	×	[53, 54]

*Note:* Automated reconstruction tools are required for the generation of a draft metabolic network during the initial step of reconstructing the systems level metabolism of an organism. Different tools require different inputs and utilize different biochemical reference databases, and some also facilitate constraint-based analysis. Tick mark indicates a feature present within the software/tool while a cross indicates the absence of a feature.

Metabolic reconstructions often require extensive manual curation to ensure they best represent the known metabolic capabilities of an organism drawn from the genome annotation and literature. Despite increasingly more sophisticated algorithms implemented in reconstruction tools to maximize gap filling and the quality of automated metabolic reconstructions [59–61], manual curation efforts are still highly recommended to ensure a GEM is of the highest quality [62]. Following the protocol detailed by Thiele & Palsson [63], common stages in this curation process include updating metabolite and reaction identifiers to a universal format, such as those detailed on the BiGG database [55], gap filling by manual cross-checking with reference databases, literature searches and the identification of potential candidate orthologous genes which could fill these gaps [59].

Metabolic reconstructions are ultimately tools designed for researchers to utilize in their own metabolic research, and therefore platforms storing these models for ease of access are important. The BiGG database [55] currently contains 108 highly curated GEMs for both prokaryotic and eukaryotic organisms all with standardized identifiers for metabolites and reactions which are often integrated into other newly reconstructed GEMs (Table 1). Others include MEMOSys [64] which tracks changes made to GEMs available on the platform and MetExplore [65] which is primarily designed for collaboration.

Each of these tools has individual strengths and weaknesses, which must be taken into consideration. A study looking at differences between automated reconstructions and manually curated networks for *Lactobacillus plantarum* and *Bordetella pertussis* identified that while RAVEN performed the best for maintaining gene similarity, it maintained reaction similarity the least [66]. The Model SEED, Pathway Tools and *merlin* generated networks with the largest proportion of genes not present in the manually curated models [66], further emphasizing the need for manual curation during model reconstruction. MetaDraft maintained metabolite similarity the best and Model SEED, CarveMe and Pathway Tools generated reconstructions the fastest [66]. It is also important to consider the different templates these tools are utilizing for the construction of the biomass reaction and whether this is consistent with a Gram-positive or Gram-negative organism.

### Constraint-based analysis approaches

**Figure 2:**
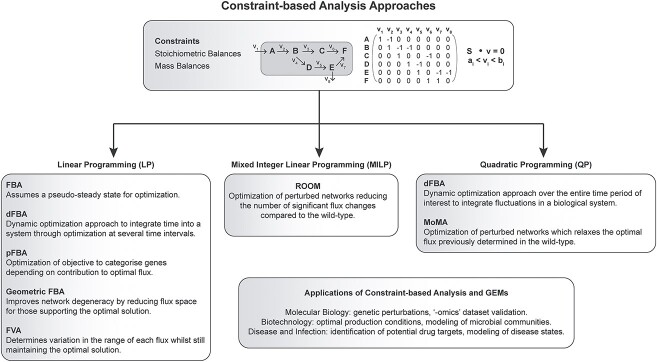
Constraint-based analysis approaches for metabolic reconstruction simulations. Metabolic reconstructions are analyzed by imposing stoichiometric and mass balance constraints to generate phenotypic predictions. Linear programming (LP) generally lends itself to Flux Balance Analysis (FBA) which can be expanded beyond the classical steady-state assumption to become dynamic by integrating time (dFBA), to integrate the underlying geometry of the metabolic network (geometric FBA) and to include gene categorization (pFBA). Flux Variability Analysis (FVA) is also achieved through LP to determine the flux range of reactions in a network. Mixed Integer Linear Programming (MILP) is typically applied to the analysis of genetically perturbed networks (i.e. ROOM). Quadratic Programming (QP) can also be used to analyze perturbed networks (i.e. MoMA) but can also expand FBA to integrate time in a dynamic fashion (dFBA).

Fluxes across reactions and metabolites in a metabolic reconstruction can be quantified whereby a specific objective function is optimized while satisfying imposed constraints [67–70]. Various constraint-based analysis approaches (Figure 2) have been developed and implemented for the analysis of GEMs, some of which are summarized below.

#### Flux balance analysis (FBA)

FBA is the first and by far the most common constraint-based analysis approach for analyzing biological networks at the systems level [69, 71]. FBA imposes reaction stoichiometry and mass balance constraints, both of which are simple to implement [71]. A particular objective function can be simulated *in silico*, such as optimizing biomass production to predict growth phenotypes [72, 73], using FBA whereby the fluxes in the reconstruction must satisfy the stoichiometric and mass balance constraints in an assumed steady state [74]. The steady state allows the network to be mathematically represented through a series of linear equations which can be solved through linear programming [75]. FBA has been integrated with GEMs to address several biological questions, such as *in silico* gene deletions [76–78], characterization of minimal media conditions [78, 79] and the identification of potential drug targets [12, 79]. However, while the simplicity of FBA allows for its widespread implementation, it does have its limitations. The quality of predictions made through FBA is wholly dependent on the curation efforts during the reconstruction phase of creating a GEM; the presence of gaps or dead-end metabolites in a network will increase the unreliability of these predictions [80, 81].

#### Dynamic FBA (dFBA)

The steady state assumed in FBA is often not applicable to biological systems, whereby the environment will inevitably fluctuate over time. FBA can be extended to describe these dynamics by integrating a GEM with external metabolites to determine intracellular fluxes [82]. dFBA is performed either through a dynamic optimization approach (DOA) or a static optimization approach (SOA). Like FBA, the SOA uses linear programming to optimize an objective over time by repeatedly solving for this objective in separate time intervals [83]. In contrast, DOA utilizes non-linear programming to optimize for an objective over the entire time period of interest to obtain a single solution [82, 83]. dFBA has successfully been applied to several biological problems, including the modeling of human disease phenotypes [84], the modeling of microbial communities [3, 9, 85] and the optimization of biotechnological processes [86, 87].

#### Parsimonious FBA (pFBA)

pFBA, or parsimonious enzyme usage FBA, utilizes linear programming to classify genes present in a reconstruction depending on their contribution to the optimal solution and their associated flux [88]. pFBA is predominantly used to validate the expression of genes and proteins determined through transcriptomics or proteomics studies and their potential downstream metabolic effects [88, 89].

#### Geometric FBA

The optimal value for an objective function determined through FBA can often be achieved by multiple different solutions, known as network degeneracy [68, 75]. Ideally, the solution space in which these fluxes are distributed to support the optimal solution needs to be reduced and this can be achieved by considering the geometry underpinning the network [90]. Utilizing a geometric FBA approach can aid in the identification of particular metabolic pathways contributing to differences in model predictions in different simulations [91].

#### Minimization of metabolic adjustment (MoMA)

GEMs are often used to assess the effect of genetic perturbation on phenotypic predictions, with the assumption that these altered networks still represent the optimal metabolic state [92]. However, this is not representative of *in vitro* mutants. MoMA accounts for this discrepancy by relaxing the optimal flux distribution and minimizing the Euclidean distance between the wild-type and mutant flux distribution while still implementing the same constraints as FBA using quadratic programming [92]. MoMA has been used in metabolic engineering to identify optimal production conditions for products of biotechnological interest [93, 94].

#### Regulatory on/off minimization (ROOM) of metabolic flux changes

Similar to MoMA, ROOM is also a method used to make phenotypic predictions with GEMs following gene deletions [95]. ROOM differs from MoMA in that it identifies a flux distribution in a model experiencing a gene deletion which satisfies constraints while minimizing the number of significant flux changes in comparison to the original model [95], and also uses mixed integer linear programming.

#### Flux variability analysis (FVA)

This analysis approach works on the same principles as FBA, using the same stoichiometric and mass balance constraints. For each reaction in a reconstruction, FVA determines the minimum and maximum flux value which still satisfies the constraints and results in the optimal solution, thereby identifying the flux variability [75], accounting for network degeneracy explained previously. A similar result has also been described utilizing an approach whereby the minimum set of reactions which fulfill the optimal solution to an objective are identified [96]. FVA is commonly used to assess how well a network responds to perturbations, also referred to as robustness, but other applications include identifying optimal production conditions for a product of interest [97, 98].

### Constraint-based modeling tools

Various tools exist to perform constraint-based analysis on metabolic reconstructions (Table 2). Toolboxes, which are installed as libraries in other commonly used analysis or visualization software [111], are by far the most commonly used and comprehensive in terms of the type of analysis that can be performed within them. The two most common available toolboxes are COBRA [99] and RAVEN [45], both of which can be installed as libraries in MATLAB. The COBRA toolbox has algorithms to perform all the different types of analyses described above, while RAVEN can be used for most with the exception of pFBA, geometric FBA and ROOM [45, 99]. In addition, both toolboxes can be used to aid the reconstruction process, including functions for gap filling and updating identifiers from other databases. Other available toolboxes include FluxAnalyzer [102], SNA [103], FBA-SimVis [104] and MetaFlux [80]. These toolboxes are capable of performing basic FBA and FVA, though are very limited in types of the other constraint-based analysis which can be implemented, unlike the COBRA and RAVEN toolboxes which have a far wider range of available analysis algorithms (Table 2).

**Table 2:** Constraint-based analysis tools

Tool	FBA	dFBA	pFBA	Geometric FBA	MoMA	ROOM	FVA	Reference
COBRA	✓	✓	✓	✓	✓	✓	✓	[99]
RAVEN	✓	✓	×	×	✓	×	✓	[45]
OptFlux, FASIMU	✓	×	×	×	✓	✓	✓	[100, 101]
SBRT, MetaFluxNet, SurreyFBA, GEMSiRV, FluxAnalyzer, SNA, FBA-SimVis, FAME, Acorn, GSMN-TB	✓	×	×	×	×	×	✓	[50, 51, 102–109]
MicrobesFlux	✓	✓	×	×	×	×	×	[110]
MetaFlux, Model SEED	✓	×	×	×	×	×	×	[44, 80]

*Note:* All of the constraint-based analysis tools available for metabolic reconstruction simulations are capable of performing standard Flux Balance Analysis (FBA), but there are also several tools available to perform more advanced analyses which are capable of analyzing perturbed networks. The COBRA toolbox [99] is the most comprehensive of the tools described in this review. A tick mark indicates a feature present within the software/tool while a cross indicates the absence of a feature.

While toolboxes are installed within other mathematical software, there are also pieces of software which can be installed independently for analysis of metabolic reconstructions. While not as comprehensive as COBRA or RAVEN, OptFlux [100] and FASIMU [101] perform basic FBA, FVA, MoMA and ROOM. Other types of stand-alone software packages include the Systems Biology Research Tool (SBRT) [105], MetaFluxNet [106], SurreyFBA [107] and GEMSiRV [51].

Both toolboxes and independent software packages require some programming experience, but there are several web-based platforms which do not require any prior programming knowledge and are very easy to use. These platforms are more rendered for model reconstruction, such as the Model SEED [44] discussed previously, so do have limited capabilities though all can perform basic FBA. However, MicrobesFlux [110] can also perform dFBA and FAME [50], Acorn [108] and GSMN-TB [109] can run FVA.

In recent years, there has been a huge increase in the development of tools to analyze the metabolism of microbial communities including the gut microbiota [112], determining metabolic limitations of such communities [113] and predicting microbial abundances within consortia [114]. Modeling and analysis of metabolic communities is challenging in terms of selecting an appropriate objective to account for trade-offs between the different species in the consortia and the community growth rate [9] as well as logistical challenges such as the integration of multiple reconstructions for each species in the community [113]. Community FBA (cFBA) is a methodology developed for such purposes [113], which integrates individual microbial GEMs and details metabolite exchanges between the species in the community [113], but other available tools include MICOM [112], SteadyCom [114] and metaGEM [115] which is a pipeline for metabolic reconstruction of microbial communities from metagenomes.

### Integration of ‘-omics’ data

**Figure 3:**
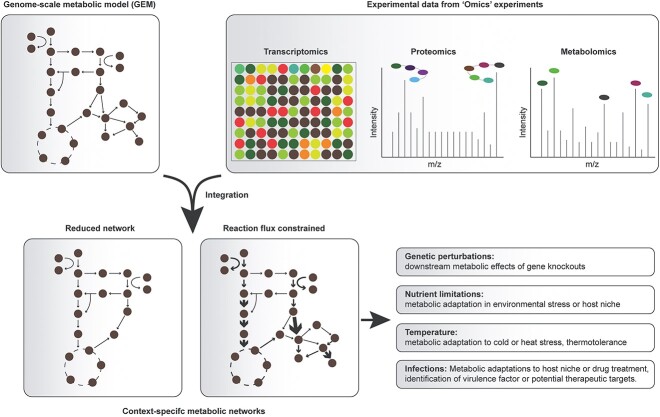
Integration of ‘-omics’ data to create context-specific models. Context-specific models describe the metabolism of an organism in two particular conditions, providing valuable platforms to study the metabolic adaptations occurring in an organism in a particular condition. Mapping gene or protein expression to the gene-protein-reaction associations present in metabolic reconstructions can define which reactions are active and inactive between two different conditions while metabolomics data can provide insight into metabolic use requirements. Context-specific models have several applications in biology, including investigating the effect of environmental and genetic perturbations in microbial strains.

GEMs can be expanded through the integration of transcriptomics, proteomics and metabolomics data to create context-specific models (Figure 3), describing the metabolism of an organism in a particular environment. The majority of research involving the reconstruction of context-specific models refers to those describing the metabolism of a particular cell line or tissue [116–118], or to human models to identify potential therapeutic targets [119, 120]. However, context-specific microbial metabolic models also have several applications.

GEMs contain gene-protein-reaction (GPR) associations, and therefore gene expression data obtained through transcriptomic experiments can be integrated into networks by adjusting reaction fluxes. Gene expression data are mapped onto GPR associations to define inactive and active reactions in the condition of interest, and from this definition context-specific models are extracted [121]. Several algorithms have been developed to integrate such data with metabolic networks, but the most common are the Gene Inactivity Moderated by Metabolism and Expression (GIMME) algorithm [20] which can be implemented from within the COBRA or RAVEN toolboxes [45, 99] and the integrative Metabolic Analysis Tool (iMAT) [122]. GIMME uses quantitative gene expression data, such as differential gene expression datasets, along with an objective function [20] to alter reconstructions to represent the condition in which the transcriptomics data were collected. iMAT can integrate functional data, either from a transcriptomics or proteomics experiment, with a metabolic network to create a map in which the metabolic state of an organism in a particular condition can be visualized [122]. Interestingly, it has been shown that the accuracy of flux predictions is comparable for models integrated with transcriptomics or proteomics data [123].

To ensure that context-specific models are representative of the organism’s metabolism in a particular context, it is vital to evaluate expression scores while mapping to GPR associations [22, 121]. Thresholds for these expression scores must be determined carefully to ensure predictive accuracy of these reconstructions. Several algorithms have been developed for this purpose, such as single-sample Gene Set Enrichment Analysis (ssGSEA) which can be combined with GIMME [124]. Consideration of such expression scores allows housekeeping genes, which will be expressed in low quantities, to be kept in the final reconstruction [22]. There are also developed techniques to utilize transcriptomics data to expand metabolic reconstructions to include information regarding regulatory interactions for the reconstruction of transcriptional regulatory networks (TRNs) [125].

The GIMME algorithm can also be extended to include metabolomics: the Gene Inactivation Moderated by Metabolism, Metabolomics and Expression (GIM3E) algorithm [126]. Integration of metabolomics data into metabolic reconstructions relates the structural properties of the network to quantitatively determined metabolite levels [126]. GIM3E determines metabolite use requirements from metabolomics data to ensure that necessary metabolites are accounted for in the reconstruction and carry flux to optimize the objective function [126], and acts as a further constraint alongside the gene expression data. Integration of such data reduces the number of alternate optimal solutions following constraint-based analysis [67]. Other tools, such as Relative Expression and Metabolomics Integration (REMI) [67], utilize exo-metabolomics data to integrate metabolic abundance data into reconstructions to maximize metabolite concentrations in context-specific models [21].

Integration of microbial GEMs with ‘-omics’ data can enhance our understanding of microbial metabolism during infection such as deciphering the metabolic changes induced in pathogens by antibiotic treatment [127], exploring changes in growth rate experienced by microbes during adaptation [21] and identifying potential virulence factors [15, 16]. Context-specific models can also have biotechnological applications including analyzing the thermotolerance of biomass degrading fungi and yeasts with potential use in the food and pharmaceutical industry [128].

For models of *Escherichia coli* and *Saccharomyces cerevisiae* integrated with transcriptomics data, pFBA was the most accurate at predicting reaction fluxes compared with experimentally measured fluxes [123]. However, metabolomics data are sparce, and the small amount of experimental data available is likely to introduce biases [123] when comparing different methods for the generation of context-specific models.

### Investigating genetic perturbations

Genome-scale metabolic models are a valuable tool to investigate the lethality and downstream metabolic effects of gene knockouts or mutations in species [80, 92, 95, 129], which is not always feasible, both practically and financially, in a laboratory setting.

#### Identifying essential genes

**Figure 4:**
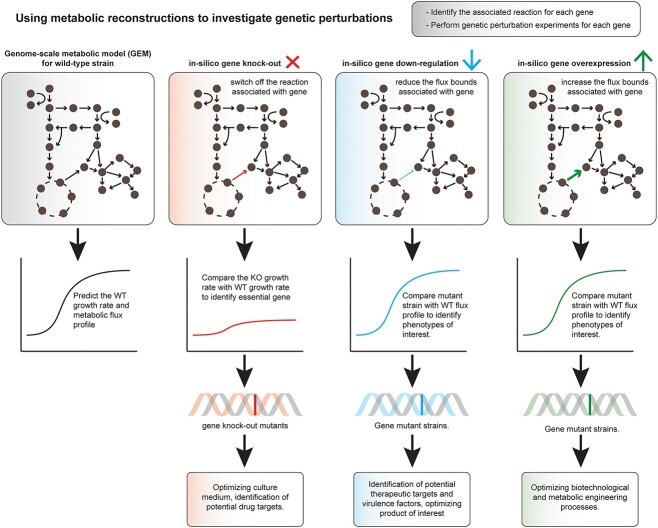
Investigating the effect genetic perturbations have on the metabolism of an organism. Genetically perturbed microbial strains, including single or double gene knockouts (KOs) and gene knockdowns, can be investigated using GEMs. *In silico* single gene KOs, performed by making all reactions controlled by an individual gene inactive, can identify candidate essential genes for further investigation *in vitro* to optimize culture medium or as potential therapeutic targets. Mutant strains can be created *in silico* by incorporating gene KOs and/or gene knockdowns, by inactivating and reducing the flux of reactions controlled by a gene(s), respectively. Optimization of mutant strains *in silico* can predict mutant phenotypes which can be further confirmed in the laboratory for the identification of potential drug targets, virulence factors and metabolic engineering purposes. By increasing the flux of reactions associated with an individual gene, *in silico* genetic overexpression can be used in metabolic engineering to predict candidate genes which increase the yield of a product of interest. The growth rate of mutant strains can be compared to the predicted growth of the wild-type model to elucidate any phenotypic effects induced by the genetic perturbation.

Genome-scale essentiality studies have become increasingly popular in the last 20 years, but they are expensive and not always feasible, particularly for less well-studied species. Such datasets are often used to validate phenotypic predictions generated from model reconstructions [15, 130, 131]. However, context-specific models can be utilized to identify potential essential genes in simulated conditions of interest (Figure 4). To simulate the knockout (KO) of a particular gene, all associated reactions are switched off and the model is simulated to optimal growth; if this predicted growth rate is zero then the gene is considered essential. It has been shown that essential genes predicted by highly curated and validated models in this approach are highly accurate, and these reconstructions can be used for the identification of potential drug or therapeutic targets [19, 132–134].

#### Investigating mutant strain phenotypes

Generating mutant bacterial strains, gene knockdowns or knockouts, can be challenging and elucidating any downstream effects can be even more challenging. Creating reconstructions of mutant strains *in silico* can be particularly helpful to determine the effects this genetic perturbation has on metabolism. For example, simulation of *in silico* single and double mutant strains of *Mycoplasma pneumonia*, using an experimentally validated GEM, revealed that biomass synthesis in this pathogen has very limited adaptability [135]. Metabolic modeling can also be used as a platform to investigate the mechanisms underlying known mutant phenotypes (Figure 4); however this is currently rarely applied to microbial models and instead has been used in models of eukaryotes, such as the model organism *Neurospora* [136].

#### Examining gene overexpression

Strain-design algorithms can be implemented with GEMs in metabolic engineering approaches to identify optimal conditions for the maximal production of a particular metabolite of interest. Such approaches have been implemented to identify that the overexpression of genes encoding the enzymes acetyl-CoA carboxylase and branched-chain α-keto acid dehydrogenase in *Streptomyces coelicolor* increase antibiotic production [137]. Similar approaches have also been used to optimize rapamycin and ascomycin production by *Streptomyces hygroscopicus* [5, 7]. Guided strain design can also utilize FVA to make similar predictions for products of industrial interest, such as increasing the production of isoprene by overexpressing glycolysis and cofactor biosynthetic genes [6] (Figure 4).

### Investigating environmental perturbations

**Figure 5:**
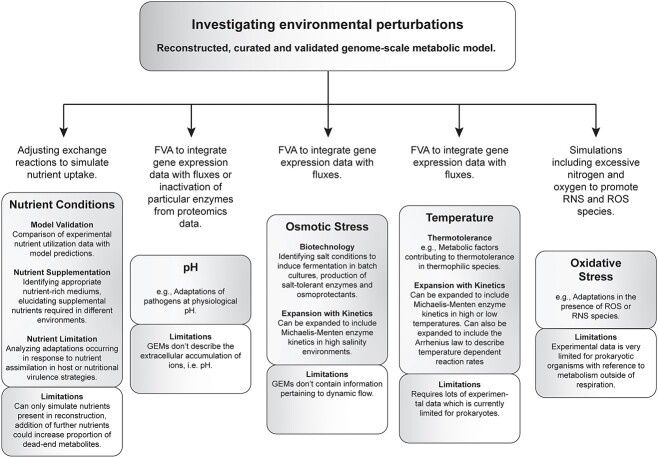
Investigating environmental perturbations using metabolic reconstructions. Context-specific genome-scale metabolic models are valuable platforms to study the metabolic adaptations occurring in an organism in response to different environmental perturbations, including altering nutritional environments, pH, osmotic stress, temperature changes and oxidative stress. These perturbations are predominantly studied through the integration of transcriptomics and proteomics data, but stoichiometric reconstructions can also be expanded to include further kinetic constraints to create more predictive models in regards to environmental perturbations, particularly osmotic stress and temperature changes.

Context-specific models can be an invaluable tool when studying the metabolic adaptations microbes employ to various stresses encountered in the environment (Figure 5), and can help elucidate the mechanisms underlying such stress responses in both commensal and pathogenic microbial species.

#### Nutrient limitation and supplementation

Nutrients available in an organism’s environment can vary depending on whether this environment is aerobic or anaerobic, the population of different microbial species also present and if it represents a niche within a multicellular host. Exchange reactions describing the uptake of a metabolite from the extracellular to cytosolic compartment can be used to simulate the nutrient conditions of interest [138]. The ability to alter the nutrient availability during constraint-based analysis allows the identification of media compositions for a variety of purposes, which can be replicated experimentally, such as a nutrient-rich medium to identify essential genes in *Mycobacterium tuberculosis* [139], the identification of supplemental nutrients to support the anaerobic growth of *Parageobacillus thermoglucosidasius* [140] and examining how lysine supplementation triggers underground polyamine metabolism as an antioxidant strategy [141]. Analysis of the utilization of multiple nutrients is frequently used as a validation step during GEM reconstruction through comparison with experimental data [77, 130, 142–144].

Pathogenic and symbiotic microbes often encounter nutrient limitations in the host niche, and must adapt their metabolism accordingly in regards to nutrient assimilation [145]. In response to infection by bacterial pathogens, mammalian hosts often sequester essential nutrients in an attempt to prevent bacterial growth, yet several of these pathogens have developed techniques to subvert these mechanisms, which is often referred to as a nutritional virulence strategy [146]. Metabolic reconstructions can be used to analyze the metabolic adaptations which occur in bacterial pathogens to best survive within the host niche by simulating these limiting nutrient conditions encountered in these environments [147–149].

#### pH

Microbes encounter changes in pH in several environments, such as during fermentation, as part of the gut microbiota and in the soil following rainfall. While GEMs do not contain ion channels, they do include the presence of charged ions as metabolites, including protons, with corresponding exchange reactions between the extracellular and cytosolic space. However, metabolic reconstructions do not contain information on the extracellular accumulation of such ions which would describe the change in environmental pH. Integration of ‘-omics’ data with metabolic models can still lead to the creation of context-specific models encapsulating the metabolism of an organism at a particular pH. The integration of proteomics data for *Enterococcus faecalis* grown at physiological pH revealed mechanisms the pathogen undergoes to grow at the pH encountered in a human host [150]. Use of experimental data describing enzymatic function at a particular pH can also be integrated with the GPR associations in a reconstruction, though this relies extensively on specific experimental data of the kind of which is extremely limited in the literature at present [151].

#### Osmotic stress

Studying the metabolic profile of halophilic bacteria has revealed several adaptations that contribute to the survival of such microorganisms in response to osmotic stress. Metabolic reconstructions of the well-characterized halophilic genuses *Chromohalobacter* and *Microbacterium* have successfully reproduced phenotypes displaying the osmoadaptation of these species, predominantly focused on the accumulation of osmoprotectants and production of salt-tolerant enzymes which have applications in biotechnology [18, 152–154]. Microbes also encounter osmotic stress in artificial environments, such as batch cultures. Studies using metabolic reconstructions of *E. coli* identified the optimal salt concentration in batch cultures to induce a switch from aerobic to fermentative pathways [17]. Typical stoichiometric reconstructions can be expanded to also include reaction kinetics, such as Michaelis–Menten kinetics of enzymes, to create kinetic models which are more mechanistic representations of a system than metabolic reconstructions analyzed through FBA [155]. Kinetic models are becoming increasingly applied to studying the effects enzyme kinetics in high salinity environments have on downstream metabolism, though currently these approaches have only been described in yeast [155].

#### Temperature

The majority of temperature-specific metabolic models are created through the integration of transcriptomics data of organisms growing at two different temperatures *in vitro*. The integration of ‘-omics’ data to create temperature-specific models is predominantly achieved using FVA to align flux and expression ratios [156]. Metabolic factors contributing to thermotolerance have been investigated using temperature-specific reconstructions of the fungus *Kluyveromyces marxianus* [128] and the Antarctic bacterium *Pseudoalteromonas haloplanktis* [157]. Enzyme structure and function can be affected by temperature fluctuations. Kinetic models are more typically associated with studying temperature responses in different organisms. The Arrhenius law describes how reaction rates are dependent on temperature, and using this law in kinetic models has been applied to studying temperature adaptations in eukaryotes, including yeast and plants [158]. However, the expansion of stoichiometric-based reconstructions with Michaelis–Menten dynamics and mass-action kinetics can also be used to study the bacterial metabolic response to fluctuations in temperature [159]. The recently published GECKO toolbox could enhance the generation of temperature-specific microbial reconstructions through providing a platform to integrate enzyme constraints elucidated through proteomics data [160].

#### Oxidative stress

Context-specific metabolic models detailing microbial metabolism in conditions of oxidative stress are relatively limited compared to the other environmental perturbations described. However, the limited examples available do provide insights into the metabolic pathways vital for metabolic adaptations during oxidative stress. Models of the gut bacterium *Enterococcus* have identified that the metabolism of reactive oxygen species induced by cancer treatments is highly linked with the metabolism of folate [161], while simulating an environment conducive to the production of reactive nitrogen species in *Rhodococcus* predicted a metabolic reroute through the pentose phosphate pathway [162].

## CONCLUSION

GEMs are becoming an increasingly valuable tool to study how the metabolism of microorganisms facilitates adaptation to different environmental perturbations. Metabolic reconstructions of a microbial species can be generated from a genome annotation using several different tools, with the COBRA Toolbox [99], RAVEN Toolbox [45] and the Model SEED [44] being the most comprehensive. Other tools can generate these reconstructions from an unannotated sequence, such as CarveMe [47], while others can utilize other published GEMs as templates, including AuReMe [49], GEMSiRV [51] and MetaDraft [54].

While multiple genetic or environmental perturbations can be studied using metabolic reconstructions, the type of constraint-based analysis applied to the study of these different perturbations, and the tool chosen for this analysis, needs to be carefully selected in order to get the most accurate predictions. The most common type of constraint-based analysis applied to predict fluxes across microbial metabolic networks is FBA, which can be expanded to account for fluctuating dynamics across time in dFBA or for network topology in geometric FBA. Other approaches can assess the effects of genetic or environmental perturbation on a system, including MoMA [92], ROOM [95] and FVA [75, 96]. Both FBA and FVA can be implemented in almost all available constraint-based analysis tools. The COBRA Toolbox can implement all the constraint-based analysis described in this study [99]. All the remaining tools are limited to the implementation of FBA and FVA with the exception of FASIMU [101] and OptFlux [100] which can implement MoMA and ROOM, and the RAVEN Toolbox [45] which is capable of MoMA and dFBA.

The study of some environmental perturbations, particularly temperature and pH, is limited with standard metabolic reconstructions, but integration with other data, such as enzyme kinetics, will greatly enhance the predictive qualities and applications of such models. The predictions generated by metabolic reconstructions provide valuable insight when designing experiments, such as the identification of promising gene or proteins of interest for adaptation studies that would otherwise have been infeasible to elucidate experimentally, either for financial or technical reasons.

**Key Points**

- Genome-scale metabolic models are valuable tools for investigating microbial metabolism at a systems-level, and are becoming increasingly utilized with the increasing speed, accuracy and accessibility of genome sequencing and annotation technologies.
- Several tools exist for the automated reconstruction of microbial metabolic networks, though manual curation is often still required to address gaps in these networks.
- Flux Balance Analysis (FBA) is the most common method to predict fluxes across reactions in metabolic reconstructions, but has limitations; therefore, several other methods have been developed to expand upon this methodology, including dynamic FBA, geometric FBA and parsimonious FBA.
- Microbes will adapt their metabolism in response to several genetic and environmental perturbations, and simulating these perturbations through the integration of ‘-omics’ datasets with metabolic reconstructions and the use of other constraint-based analysis approaches can aid in the elucidation of such adaptations.

## Data Availability

There is no additional data available. All the details are included in the article.

## Notes

**Elena Lucy Carter** is a final year PhD student at Warwick Medical School, University of Warwick, Coventry, CV4 7HL, UK. Her research interests are metabolic model reconstruction, microbial metabolic adaptation and bioinformatics.

**Chrystala Constantinidou** has been a Principal Research Fellow in Microbial Genomics at Warwick Medical School, University of Warwick. She is an expert in the field of next-generation sequencing.

**Mohammad Tauqeer Alam** is an Assistant Professor at the United Arab Emirates University, Al Ain, UAE. His research interests are metabolic modeling, microbial metabolic interactions, network analysis, bioinformatics and systems biology.
